# Development of a powerful synthetic hybrid promoter to improve the cellulase system of *Trichoderma reesei* for efficient saccharification of corncob residues

**DOI:** 10.1186/s12934-021-01727-8

**Published:** 2022-01-04

**Authors:** Yifan Wang, Ruiyan Liu, Hong Liu, Xihai Li, Linjing Shen, Weican Zhang, Xin Song, Weifeng Liu, Xiangmei Liu, Yaohua Zhong

**Affiliations:** State Key Laboratory of Microbial Technology, Institute of Microbial Technology, Shandong University, Qingdao, 266237 People’s Republic of China

**Keywords:** Synthetic hybrid promoter, *Trichoderma reesei*, *cbh1*, *cdna1*, Cellulase, Biomass bioconversion

## Abstract

**Background:**

The filamentous fungus *Trichoderma reesei* is a widely used workhorse for cellulase production in industry due to its prominent secretion capacity of extracellular cellulolytic enzymes. However, some key components are not always sufficient in this cellulase cocktail, making the conversion of cellulose-based biomass costly on the industrial scale. Development of strong and efficient promoters would enable cellulase cocktail to be optimized for bioconversion of biomass.

**Results:**

In this study, a synthetic hybrid promoter was constructed and applied to optimize the cellulolytic system of *T. reesei* for efficient saccharification towards corncob residues. Firstly, a series of 5’ truncated promoters in different lengths were established based on the strong constitutive promoter P*cdna1*. The strongest promoter amongst them was P*cdna1-3* (− 640 to − 1 bp upstream of the translation initiation codon ATG), exhibiting a 1.4-fold higher activity than that of the native *cdna1* promoter. Meanwhile, the activation region (− 821 to − 622 bp upstream of the translation initiation codon ATG and devoid of the Cre1-binding sites) of the strong inducible promoter P*cbh1* was cloned and identified to be an amplifier in initiating gene expression. Finally, this activation region was fused to the strongest promoter P*cdna1-3*, generating the novel synthetic hybrid promoter P*cc*. This engineered promoter P*cc* drove strong gene expression by displaying 1.6- and 1.8-fold stronger fluorescence intensity than P*cbh1* and P*cdna1* under the inducible condition using *egfp* as the reporter gene, respectively. Furthermore, P*cc* was applied to overexpress the *Aspergillus niger* β-glucosidase BGLA coding gene *bglA* and the native endoglucanase EG2 coding gene *eg2*, achieving 43.5-fold BGL activity and 1.2-fold EG activity increase, respectively. Ultimately, to overcome the defects of the native cellulase system in *T. reesei,* the *bglA* and *eg2* were co-overexpressed under the control of P*cc* promoter. The *bglA*-*eg2* double expression strain QPEB70 exhibited a 178% increase in total cellulase activity, whose cellulase system displayed 2.3- and 2.4-fold higher saccharification efficiency towards acid-pretreated and delignified corncob residues than the parental strain, respectively.

**Conclusions:**

The synthetic hybrid promoter P*cc* was generated and employed to improve the cellulase system of *T. reesei* by expressing specific components. Therefore, construction of synthetic hybrid promoters would allow particular cellulase genes to be expressed at desired levels, which is a viable strategy to optimize the cellulolytic enzyme system for efficient biomass bioconversion.

**Supplementary Information:**

The online version contains supplementary material available at 10.1186/s12934-021-01727-8.

## Introduction

The filamentous fungus *Trichoderma reesei* has been widely used as an industrial workhorse for cellulase production due to its extraordinary ability to produce large amounts of extracellular cellulolytic enzymes [1]. Among the cellulase enzymes secreted by this fungus, the cellobiohydrolase 1 (CBH1) accounts for more than 60% of total extracellular protein [2], thus the *cbh1* promoter (P*cbh1*) was generally used as a strong inducible promoter for gene expression [3, 4]. Besides, the endoglucanases (EGs) hydrolyzing amorphous cellulose by attacking the cellulose chains internally and randomly were found to make up 6–20% of the extracellular protein [5, 6]. Particularly, the β-glucosidase (BGL) occupying only 1% of the total extracellular protein was recognized as one of bottlenecks in the process of efficiently hydrolyzing cellulosic substrates [7, 8]. Therefore, this secretome of *T. reesei* is not always a well-proportioned cocktail for the economically feasible bioconversion of lignocellulosic biomass to produce biofuels and other bio-based products [9]. It is known that promoter controlling gene expression level is essential to both protein and metabolic engineering. In this case, it is prospective to optimize the cellulolytic enzyme system by increasing target gene expression using desired promoters in *T. reesei*. Indeed, suitable promoters have enabled desired expression levels of the specific cellulase genes, making *T. reesei* exhibit great potential to be cellulase hyper-producing strains [3, 10, 11]. Consequently, appropriate and robust promoters could offer great opportunities for the viable bioconversion of lignocellulosic biomass by conferring high levels of gene expression on specific enzymes to optimize cellulolytic enzyme system.

In *T. reesei*, many native constitutive promoters that can drive constant gene expression were used for protein production, such as promoters of the genes *gpd*A (encoding glyceraldehyde-3-phosphate dehydrogenase), *pdc* (encoding pyruvate decarboxylase), *eno* (encoding enolase), *tef* (encoding translation elongation factor 1α), and an unidentified gene *cdna1* [12–14]. Among these, P*cdna1* was generally seemed as one of the strongest constitutive promoters reported so far in *T. reesei* [15]. However, the productivity of protein expressed by P*cdna1* was not as much as that of the inducible promoter P*cbh1* [14]. In *T. reesei*, P*cbh1* was recognized as the most widely used promoter for protein production. And the glucose repression regions of P*cbh1* were well characterized [16]. Besides, the binding sites of the transcriptional activators Xyr1, Ace2 and Hap2/3/5, as well as the repressors Cre1 and Ace1, have been found to exist in the regulation region of P*cbh1* [17–21]. Especially, Cre1 inhibits the gene expression through carbon catabolite repression (CCR) in the presence of easily metabolized carbon source, such as glucose, and serves as one of the master repressors [17]. In this case, deletion of Cre1-binding sites is an efficient way to enhance P*cbh1* activity, which is also a strategy of promoter modifications. For example, four-tandem copies of the regulation region of P*cbh1*, which lacks the Cre1-binding sites but comprises the Xyr1-binding site, Ace2-binding site and the CCAAT boxes (the Hap2/3/5-binding sites), could serve as transcriptional amplifiers [22]. In addition, the replacement of the Cre1-binding sites by that of the transcriptional activators Ace2 and Hap2/3/5 also resulted a 5.5-fold P*cbh1* activity increase [21]. However, while the modified regulation region of P*cbh1* could act as an enhancer in initiating gene expression, the core region usually directs low basal activity when the regulation region is absent [23].

Beyond altering transcription factor binding sites, another choice to modify promoters is hybrid promoter construction [10]. This approach attempts to fuse two independent promoter activation sequences, usually the activation region to the core region. It is known that the *cis*-elements in promoters are modular and most of them retain their activities when broken away from the natural context, which enables the chimera to be constructed successfully [24]. In the nonconventional yeast *Yarrowia lipolytica*, a series copy of an upstream activation sequence (UAS) of the endogenous inducible promoter P_XPR2_ were fused to the core region of the constitutive promoter P_LEUM_, and in doing so the most efficient one amongst these hybrid promoters led to an eight-fold-higher promoter strength increase compared to the strong endogenous promoters [25]. In addition, the UAS of the inducible promoter P_GAL_ in *Saccharomyces cerevisiae* was fused to the different core region of constitutive promoters, P_TEF_, P_GPD_, P_CYC_ and P_LEUM_, respectively, and the hybrid promoters induced by galactose exhibited higher activity than that under the glucose condition [26]. These investigations indicated that the synthetic hybrid promoters could offer enormous potential in optimally modifying promoter. Although the synthetic hybrid promoters were extensively applied to improve promoters as a potential approach, it is still less commonly used in *T. reesei* [10].

During the process of cellulase hydrolysis, synergistic effects were considered to exist between the independent enzyme components, thus the ratio of each component in the total enzyme cocktail plays an important role in cellulolytic efficiency [27, 28]. Therefore, altering specific enzyme component and amending cellulase mixture to a propriate ratio are efficient approaches to optimize the cellulase system in hydrolyzing lignocellulose [29, 30]. In *T. reesei*, BGL was generally recognized as one of the targets to be overexpressed in optimizing the cellulase system due to its lower proportion [31, 32]. Introduction of heterogenous BGL from *Aspergillus* species in *T. reesei* directed by promoter P*cbh1* enabled the significant increase of cellulase activity and approximately 20% augment of released glucose in saccharification towards substrates corncob residue [33]. And overexpression of BGL from *Penicillium decumbens* also increased saccharification efficiency by approximately 20% towards cornstalk [29]. Furthermore, extra expression of endogenous BGL directed by a modified four-copy promoter of P*cbh1* led to at most 130% increment of filter paper activity and 29% of saccharification efficiency [32]. Besides, EG2 contributes 55% of the EG activity and when overexpressed in *T. reesei* even use its native promoter, a significant enhancement of lignocellulose hydrolyzing ability (119% toward corncob residues) was found [34, 35]. Therefore, optimization of *T. reesei* cellulase system by overexpressing specific essential enzymes directed by powerful promoters is a promising solution to improve the lignocellulose hydrolysis efficiency.

In this study, a novel synthetic hybrid promoter P*cc* was constructed by fusing the modified activation region of the strongest inducible promoter P*cbh1* to the core region of a strong constitutive promoter P*cdna1* after identifying their function. In addition, this work introduced a hybrid promoter for the first time to optimize the *T. reesei* cellulase system by overexpressing two essential cellulases BGLA and EG2. Furthermore, these two cellulases were co-overexpressed under the control of P*cc* to improve the saccharification efficiency of *T. reesei* extracellular enzymes cocktail towards two differently pretreated corncob residues. This novel hybrid promoter offered not only a new method in promoter engineering utilized in *T. reesei*, but also tremendous possibility to improve the cellulase hydrolysis ability at the transcriptional level.

## Results

### Characterization of a series of 5’ truncated promoters of *cdna1*

**Fig. 1:**
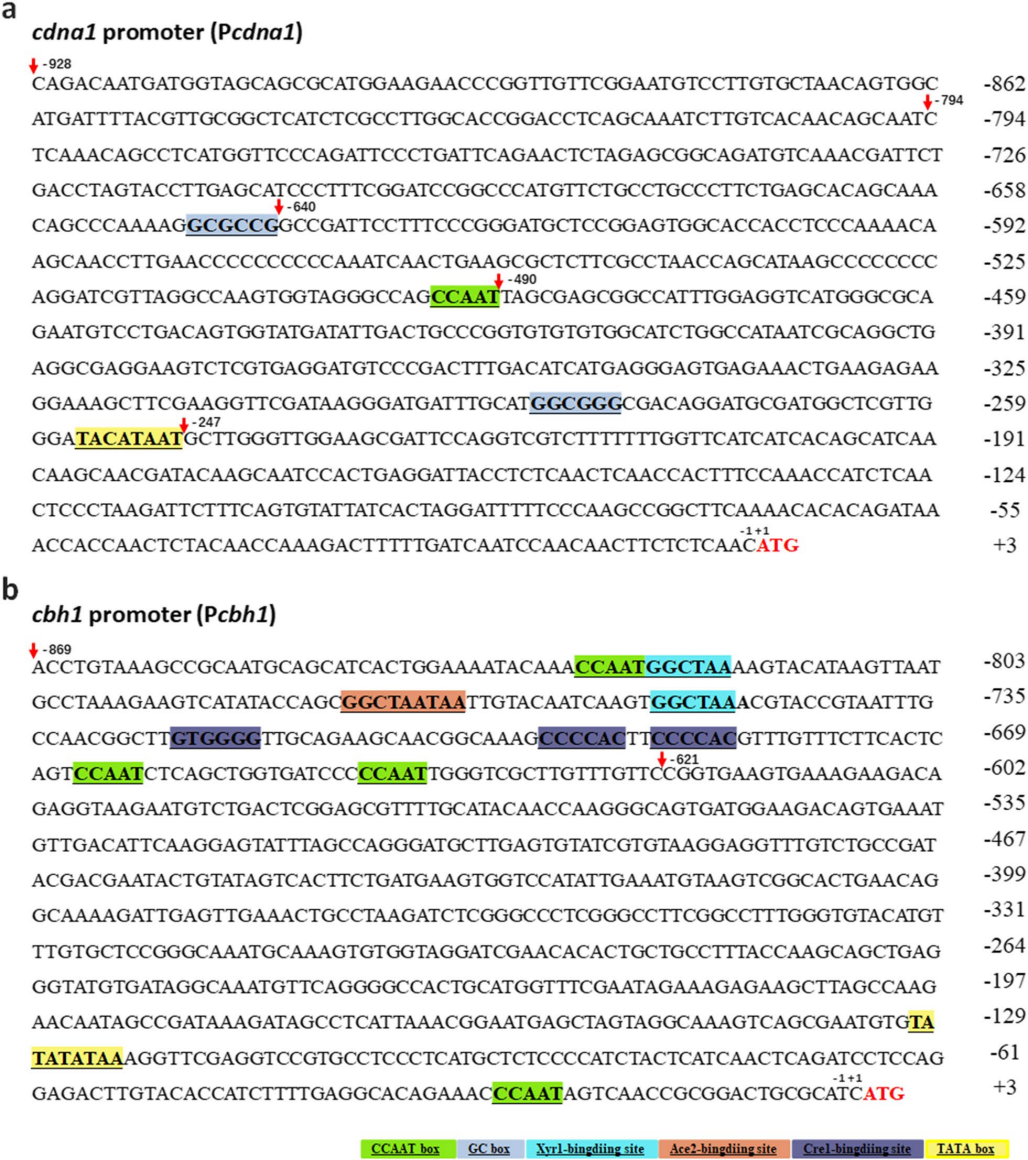
Nucleotide sequences of the P*cdna1* (**a**) and P*cbh1 *(**b**). The nucleotides ahead of A of translation initiation site (ATG) of *cdna1* and *cbh1* are numbered as − 1. Known *cis*-acting elements in P*cdna1* and mainly in the − 869 bp to − 621 bp region of P*cbh1* are shown in different color. The descriptions of elements (TATA box, CAAT box, GC box, Xyr1-binding site, Ace2-binding site and Cre1-binding site) are shown in Additional file 1: Table S1. An arrow above the sequence indicates the start point of different truncated fragments

**Fig. 2:**
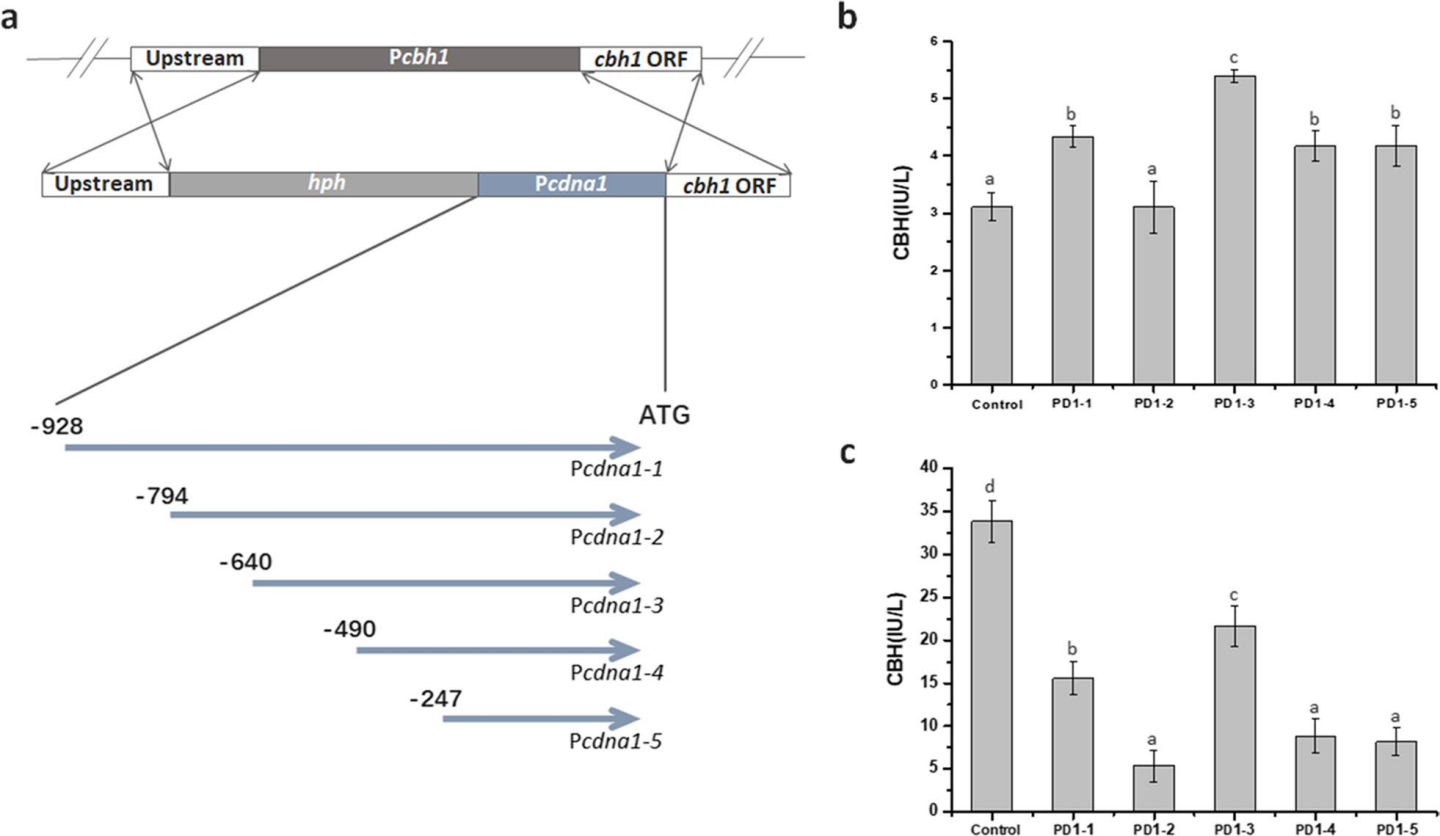
Identification of the truncated fragments of P*cdna1* fused with *cbh1* as the reporter gene. **a** Strategy of targeted replacement of P*cbh1* with the truncated fragments of P*cdna1* fused with *cbh1*. **b**, **c** CBH activity of the transformants PD1-1 (P*cdna1-1*), PD1-2 (P*cdna1-2*), PD1-3 (P*cdna1-3*), PD1-4 (P*cdna1-4*), PD1-5 (P*cdna1-5*) and the parental strain QM53 (control) cultured in GMM for 5 days (**b**) and AMM for 7 days (**c**). Data indicates the mean of three independent experiments; error bars express the standard deviations. Different lowercase letters above the bars indicate significant differences at P < 0.05

As a strong constitutive promoter in *T. reesei*, P*cdna1* has been successfully utilized to express cellulases using glucose as the carbon source [15]. Here, the 928-bp promoter region upstream of ATG of the gene *cdna1* was analyzed as shown in Fig. 1. The promoter P*cdna1* contains several core *cis*-acting elements, such as TATA box, CCAAT box and GC box, which are essential elements to initiate transcription or enhance the transcriptional level [36–38]. To functionally validate the *cdna1* promoter, a series of 5’ truncated promoters in different lengths were established based on P*cdna1*. Five 5’ truncated promoters, P*cdna1-1* (928 bp), P*cdna1-2* (794 bp), P*cdna1-3* (640 bp), P*cdna1-4* (490 bp) and P*cdna1-5* (247 bp), were fused to the reporter gene *cbh1* (coding cellobiohydrolase 1) and transformed into *T. reesei* using *hph* as the selectable marker gene, respectively (Fig. 2a). All these five truncated promoters were capable of directing CBH activity after culturing with glucose (GMM) or Avicel (AMM, Fig. 2b and c). When glucose was used as the sole carbon source, PD1-2 (*cbh1* directed by P*cdna1-2*) showed the lowest CBH activity, which was about 0.7-fold of PD1-1 (*cbh1* directed by P*cdna1-1*). However, PD1-3 (*cbh1* directed by P*cdna1-3*) exhibited the highest CBH activity, which was 1.2-fold higher than that of PD1-1 and 1.7-fold of PD1-2, indicating that the deletion of the 154-bp sequence (− 794 to − 640 bp) located between P*cdna1-2* and P*cdna1-3* enhanced promoter activity. Similar to that under the glucose condition, when the strains were cultured with Avicel, the greatest improvement of CBH activity was imparted by P*cdna1-3* (1.4-fold higher than P*cdna1-1*) while the promoter activity of P*cdna1-2* was relatively weak (0.4-fold lower than P*cdna1-1*), indicating that the *cis*-acting elements facilitating gene expression exist in the 640-bp (− 640 to − 1 bp) sequence of the promoter (P*cdna1-3*). Indeed, three kinds of essential *cis*-acting elements, CCAAT box, GC box and TATA box, were contained in P*cdna1-3* (Fig. 1a). Thus, P*cdna1-3* could be selected as the high-efficiency core region to initiate gene expression.

### Identification of the activation region of the *cbh1* promoter

The promoter of *cbh1* is known as the strongest inducible promoter in *T. reesei*, and it contains different types of transcriptional regulator-binding sites [17–21]. It has been reported that several binding sites of transcriptional activators Xyr1, Ace2 and Hap2/3/5 exist in the 248-bp (− 869 to − 622 bp) regulation region, and three Cre1-binding sites are located in the 48-bp (− 725 to − 678 bp) transcriptional repression region (Fig. 1b) [22]. Insertion of the 200-bp (− 821 to − 622 bp) regulation region without Cre1-binding sites resulted in significant higher activity than the wild-type promoter [22], demonstrating that the 200-bp (− 821 to − 622 bp) region of P*cbh1* can serve as an activation region.

**Fig. 3:**
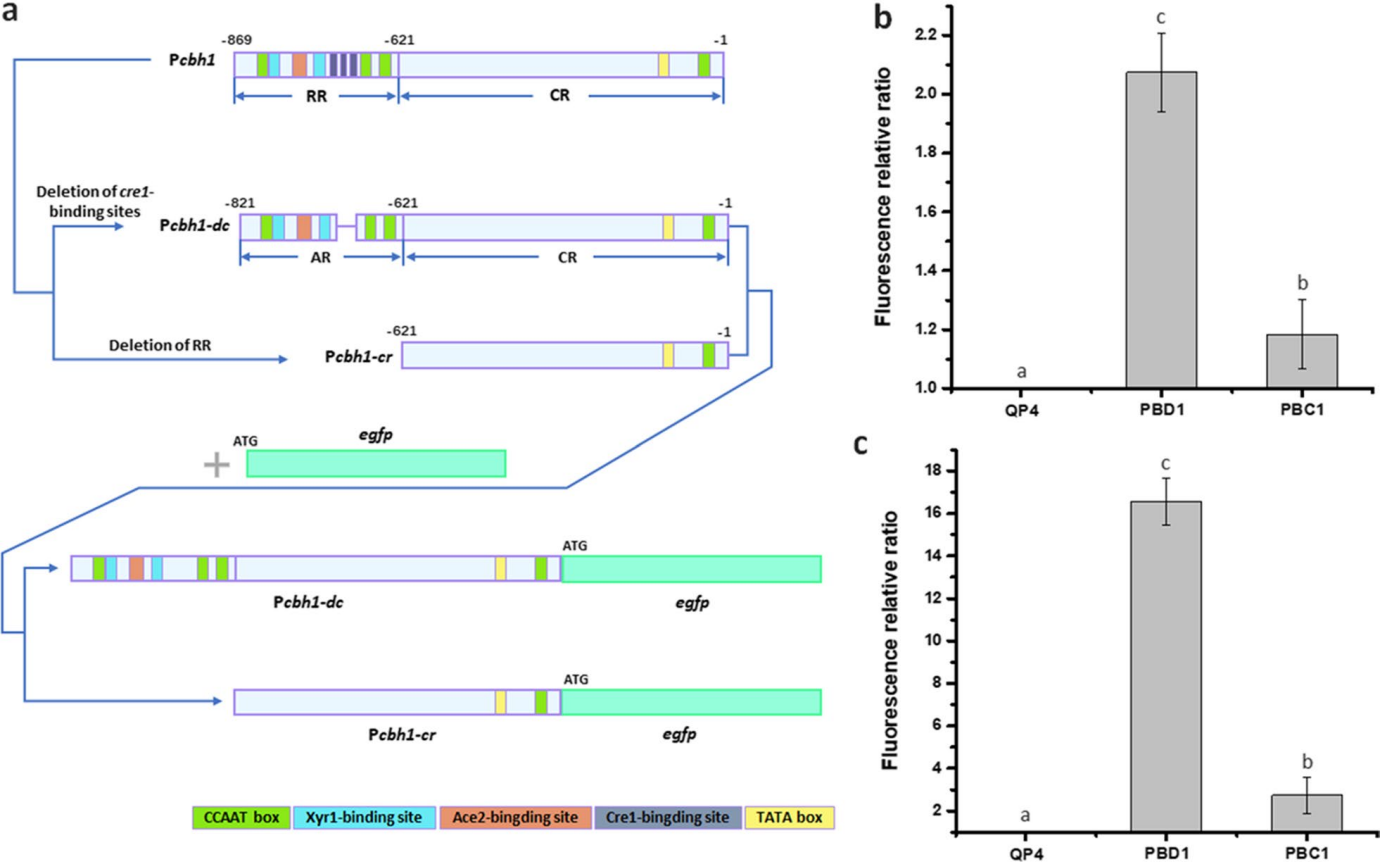
Identification of P*cbh1* activation region (AR) using *egfp* as the reporter gene. **a** Strategies of the construction of P*cbh1-dc* (P*cbh1* devoid of Cre1-binding sites) and P*cbh1-cr* (core promoter of P*cbh1*) fused with *egfp*. RR: regulation region; CR: core region; AR: activation region. **b**, **c** Fluorescence relative ratio between the transformants PBD1 (P*cbh1-dc-egfp*), PBC1 (P*cbh1-cr-egfp*) and the parental strain QP4 cultured in GMM for 2 days (**b**) and AMM for 3 days (**c**). Data indicates the mean of three independent experiments; error bars express the standard deviations. Different lowercase letters above the bars indicate significant differences at P < 0.05

Here, the 48-bp Cre1-binding sites-containing region (− 725 to − 678 bp) was deleted to construct the modified promoter P*cbh1-dc* (821 bp, containing the activation region AR and the core region CR), and the regulation region RR (− 869 to − 622 bp) was removed from P*cbh1* to construct the core-region promoter P*cbh1-cr* (621 bp) as shown in Fig. 3a. To eliminate the interference of native CBH1, the enhanced green fluorescent protein (eGFP)-encoding gene*, egfp*, was selected as the reporter gene. Then, the promoters P*cbh1-dc* and P*cbh1-cr* were fused with *egfp* to construct the corresponding cassettes, respectively (Fig. 3a). The fluorescence intensities of the transformants under the glucose (Fig. 3b) and Avicel (Fig. 3c) conditions were measured, respectively. It was found that the P*cbh1-cr* transformant PBC1 showed a barely detectable level of fluorescence intensity, which suggested that the activity of the P*cbh1* core region was relatively weak. On the contrary, the P*cbh1-dc* transformant PBD1 displayed remarkable improvement of fluorescence intensity compared to PBC1 under both glucose and Avicel conditions (Fig. 3b and c). Especially, when cultured with Avicel, PBD1 generated a 5.3-fold higher fluorescence intensity than that of PBC1 (Fig. 3c). These results demonstrated that the 200-bp (-821 to -622 bp) region of P*cbh1* could be selected as the high-efficiency activation region to facilitate gene expression.

### Construction and assessment of the hybrid promoter P*cc*

**Fig. 4:**
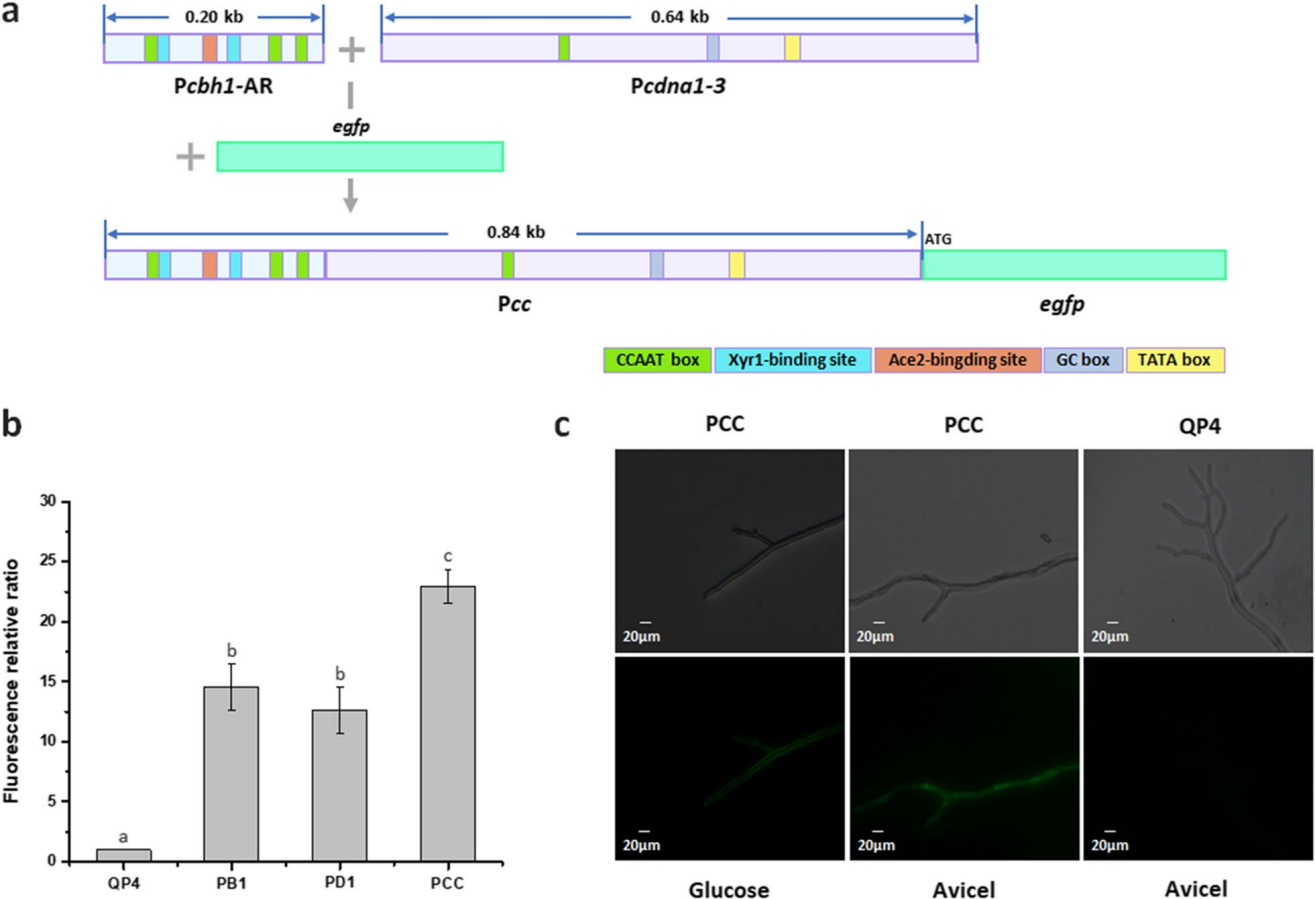
Construction of hybrid promoter P*cc*. **a** Strategies of the construction of P*cc* fused with the reporter gene *egfp*. **b** Fluorescence relative ratio between the transformants PB1 (P*cbh1-egfp*), PD1 (P*cdna1-egfp*), PCC and the parental strain QP4, respectively. Strains were cultured in AMM for 2 days. **c** The fluorescence of transformant PCC (P*cc-egfp*) and the parental strain QP4 cultured with glucose for 24 h and Avicel for 3 days were observed under the fluorescence microscope. The results shown represent one of at least three independent experiments. Data indicates the mean of three independent experiments; error bars express the standard deviations. Different lowercase letters above the bars indicate significant differences at P < 0.05

As shown above, P*cdna1-3* possesses the ability to efficiently initiate gene expression, and the 200-bp AR of P*cbh1* was determined to be an enhancer to facilitate the process, which led to the hypothesis that the combination of these two regions may generate a powerful hybrid promoter. In this regard, a novel hybrid promoter P*cc* was created by fusing the 200-bp (− 821 to − 622 bp) activation region of P*cbh1* to the 640-bp (− 640 to − 1 bp) core region of P*cdna1* (Fig. 4a). To assess the activity of P*cc*, the promoter was used to direct the expression of the reporter gene *egfp* in *T. reesei.* Meanwhile, the promoters P*cbh1* and P*cdna1* were adopted to direct the expression of *egfp* as control, respectively. Fluorescence intensity analysis was shown in Fig. 4c. As expected, the transformant PCC (*egfp* expressed by P*cc*) had the highest fluorescence intensity and reached 1.6 and 1.8 times more than the transformants PB1 (*egfp* expressed by P*cbh1*) and PD1 (*egfp* expressed by P*cdna1*) under the Avicel condition, respectively (Fig. 4b). While under the glucose condition, the fluorescence intensity of PCC was comparable to that of PD1 (data not shown). The fluorescence emission detected by fluorescence microscopy showed that the eGFP protein was produced successfully in the transformant PCC under both glucose and Avicel conditions (Fig. 4c). These results indicated that the hybrid promoter P*cc* could drive gene expression with high efficiency under both repressive and inducible conditions.

### Construction of the *T. reesei *strains overexpressing BGLA directed by P*cc*

**Fig. 5:**
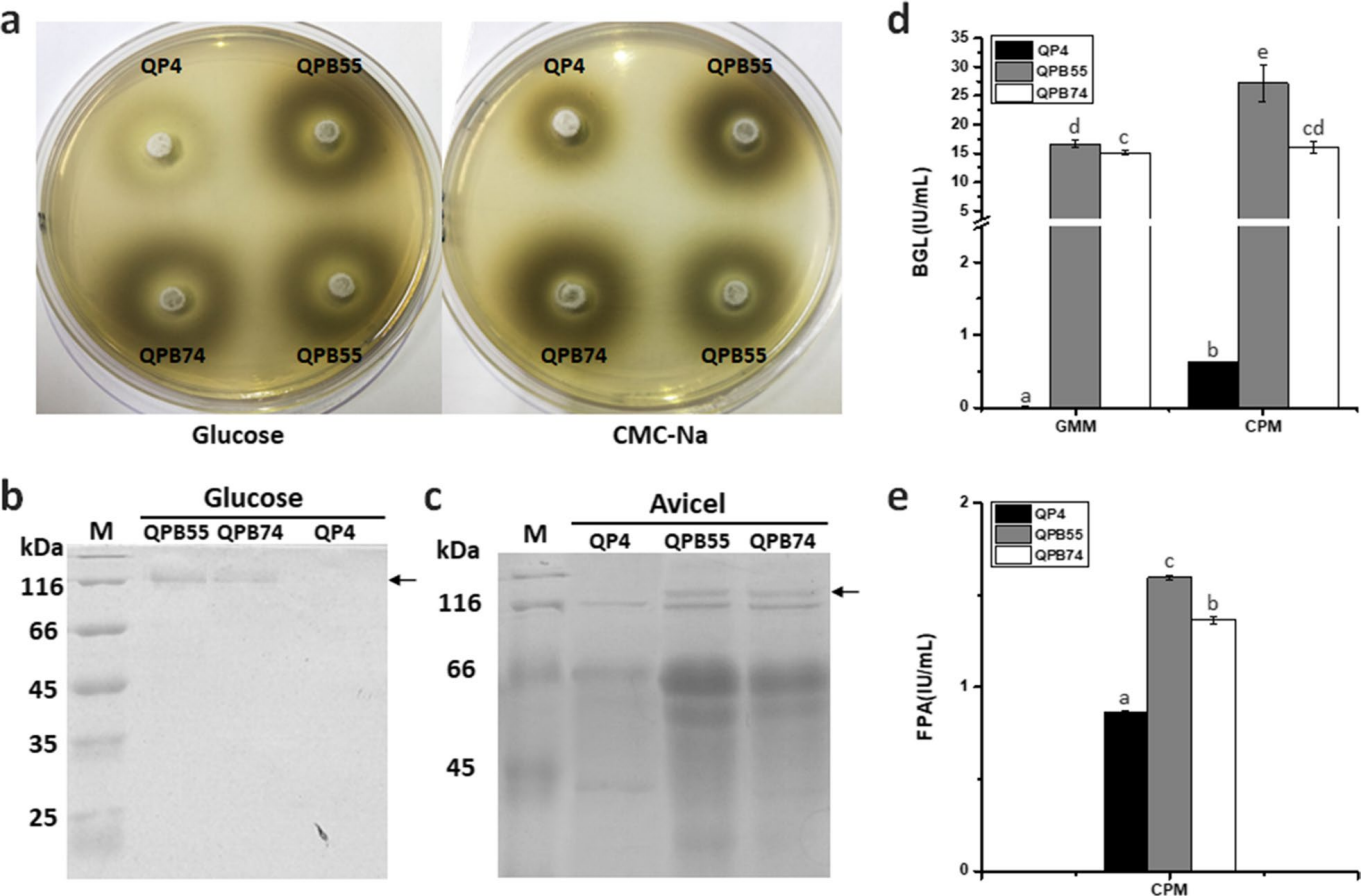
Expression of *bglA* driven by P*cc* resulted in increase of BGL activity and FPA. Transformants expressing *bglA* driven by P*cc* and the parental strain QP4 cultured on glucose-esculin plates and CMC-esculin plates at 30℃ for 2 days (**a**). SDS-PAGE analysis of the total extracellular cellulases of transformants and QP4 were shown after cultured in GMM for 5 days (**b**) and CPM for 7 days (**c**). BGLA bands were indicated by arrows. BGL activity (**d**) and FPA (**e**) of the transformants and QP4 were measured after cultured in GMM for 5 days and CPM for 7 days. Data indicates the mean of three independent experiments; error bars express the standard deviations. Different lowercase letters above the bars indicate significant differences at P < 0.05

The β-glucosidase (BGL) was recognized as one of the bottlenecks of cellulase cocktail in hydrolyzing cellulosic substrates due to its low activity in *T. reesei*. Thus, the hybrid promoter P*cc* was first attempted to direct BGL expression with the aim of optimizing the hydrolysis efficiency of cellulolytic enzyme system. Here, the *Aspergillus niger* β-glucosidase gene *bglA* was overexpressed by P*cc* in *T. reesei* using *pyrG* as the selectable marker gene. Two transformants, QPB55 and QPB74, were selected to be cultured on esculin plates to detect their BGL activity using glucose and sodium carboxymethyl cellulose (CMC-Na) as the sole carbon source, respectively (Fig. 5a). The sizes of dark halos of these transformants were much larger than that of the parental strain QP4 (an uracil auxotrophic *T. reesei* strain derived from the well-known cellulase overproducer QM9414) no matter on glucose-esculin plates or CMC-esculin plates, which indicated that the overexpression of *bglA* driven by P*cc* resulted in remarkable improvement of BGL activity. Then, fermentation of these transformants were conducted in GMM and CPM for cellulase production, respectively. SDS-PAGE analysis showed that an approximately 120-kDa band (the expected molecular weight of *A. niger* BGLA) were clearly observed under two conditions in the fermentation broths of QPB55 and QPB74, which were not present in QP4 (Fig. 5b and c). And the bands were determined to be BGLA by MS analysis (data not shown). The transformants QPB55 and QPB74 exhibited the significant increase of BGL activities (16.6 IU/mL and 15.1 IU/mL, respectively) when using glucose as the carbon source (Fig. 5d), which was in line with the results observed on esculin plates and SDS-PAGE analysis. Besides, when cultured with Avicel, QPB55 and QPB74 provided 43.5- and 25.7-fold higher BGL activities (27.2 IU/mL and 16.0 IU/mL) than that of the parental strain (0.6 IU/mL), which led to 85.2 and 58.6% enhancements of FPA (1.6 IU/mL and 1.4 IU/mL, filter paper activity) in comparison to that of QP4 (0.9 IU/mL), respectively (Fig. 5d and e). These results implied that the overexpression of *bglA* driven by the hybrid promoter P*cc* remarkedly increased the BGL activity, therefore enhancing the total cellulase activity of *T. reesei*.

### Construction of the *T. reesei* strains overexpressing EG2 directed by P*cc*

**Fig. 6:**
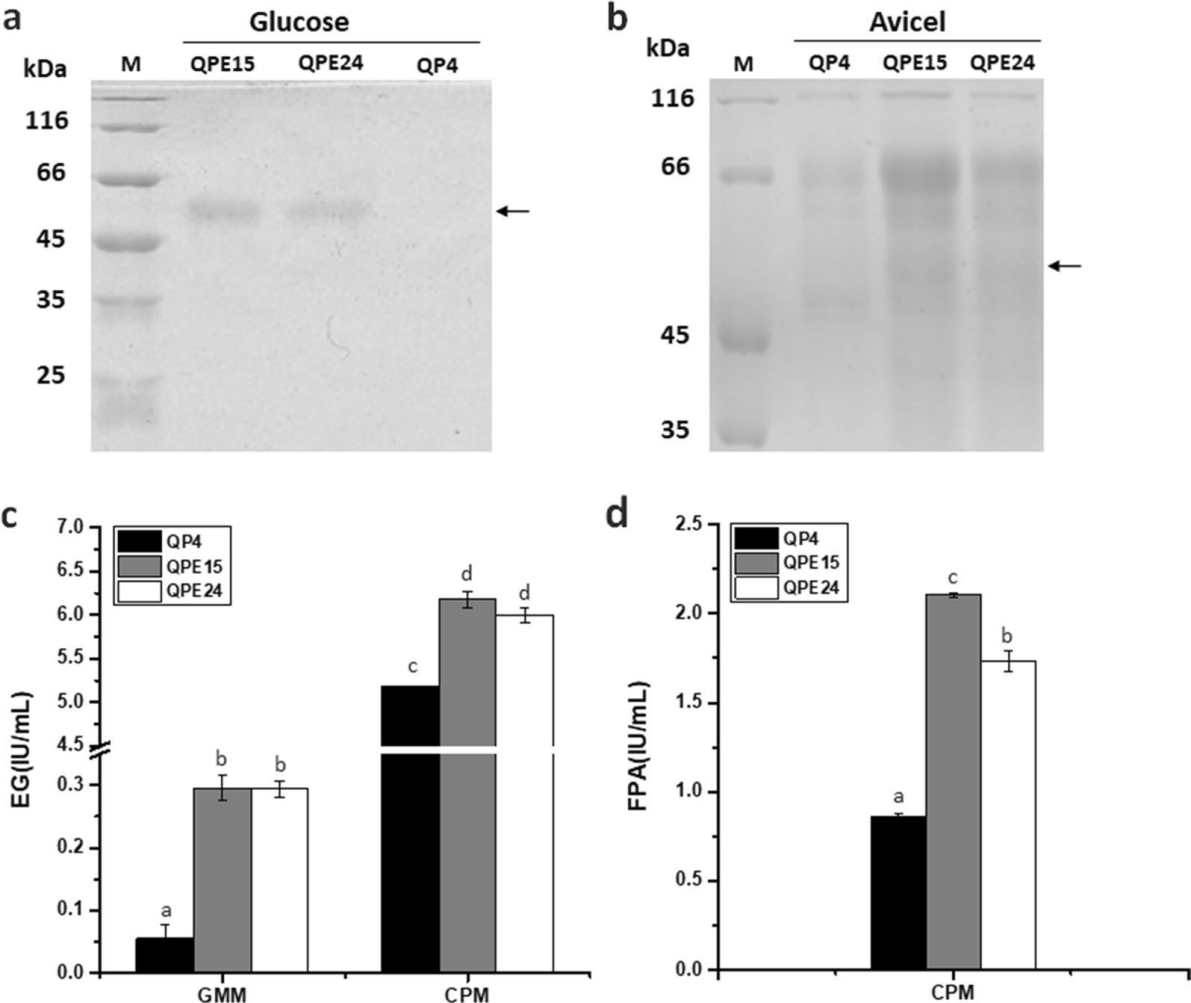
Expression of *eg2* driven by P*cc* resulted in increase of EG activity and FPA. SDS-PAGE analysis of the total extracellular cellulases of transformants and the parental strain QP4 were shown after cultured in GMM for 3 days (**a**) and CPM for 7 days (**b**). EG2 bands were indicated by arrows. EG activity (**c**) and FPA (**d**) of the transformants and QP4 were measured after cultured in GMM for 5 days and CPM for 7 days. Data indicates the mean of three independent experiments; error bars express the standard deviations. Different lowercase letters above the bars indicate significant differences at P < 0.05

The *T. reesei* EG2 has been determined to contribute the most to the endoglucanase activity, suggesting its importance in hydrolysis of cellulosic substrates [6]. Therefore, the endoglucanase gene *eg2* was also employed to be overexpressed directed by P*cc* in *T. reesei* using *pyrG* as the selectable marker gene. Then two transformants, QPE15 and QPE24, were selected and fermented in GMM and CPM, respectively. The SDS-PAGE and MS analyses demonstrated that the EG2 bands in the transformants QPE15 and QPE24 were clearly observed while the band in QP4 was not detected (cultured with glucose) or relatively weak (cultured with Avicel, Fig. 6a and b). Notably, strikingly increased EG activities (0.3 IU/mL) of these transformants were detected under the glucose condition, whereas EG activity of QP4 was considerably weak (0.06 IU/mL, Fig. 6c). While cultured with Avicel, QPE15 and QPE24 showed 19.1 and 15.7% increase of EG activities (6.2 and 6.0 IU/mL) compared to QP4 (5.2 IU/mL), leading to 2.5- and 2.0-fold higher FPA (2.1 and 1.7 IU/mL) than that of QP4 (0.9 IU/mL), respectively (Fig. 6c and d). These results indicated that overexpression of *eg2* driven by P*cc* remarkedly increased the EG activity, therefore improving the total cellulase activity of *T. reesei*. Based on the observation that the total cellulase activity of *T. reesei* can be improved by overexpressing specific cellulases directed by P*cc*, this hybrid promoter was demonstrated to be efficient in optimizing cellulase system.

### Construction of the *T. reesei *strains co-overexpressing BGLA and EG2 directed by P*cc*

**Fig. 7:**
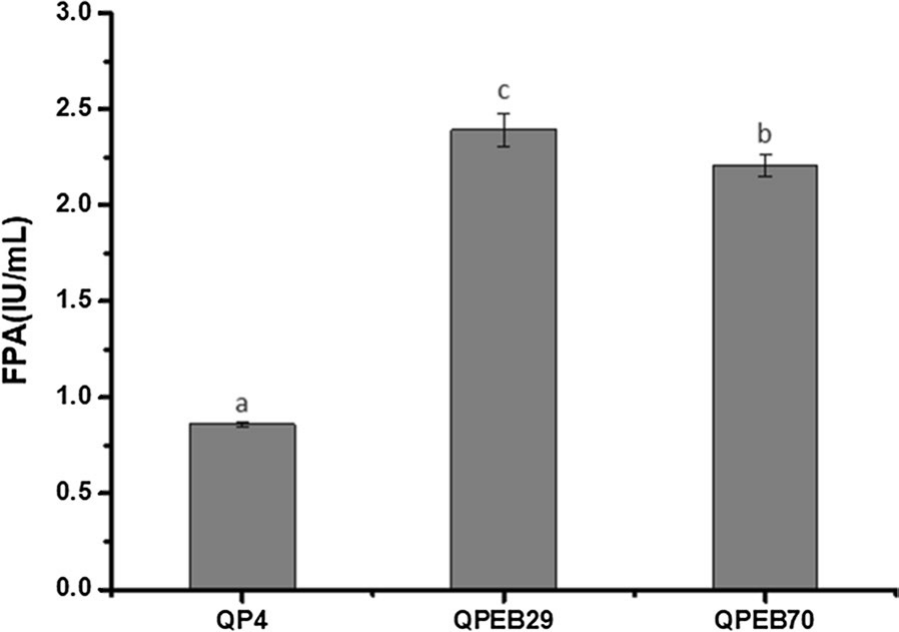
Co-overexpression of *bglA* and *eg2* driven by P*cc* in *T. reesei* resulted in increase of FPA. The transformants and the parental strain QP4 were cultured in CPM for 7 days. FPA were measured at the end of cultivation. Data indicates the mean of three independent experiments; error bars express the standard deviations. Different lowercase letters above the bars indicate significant differences at P < 0.05

Given that the individual overexpression of BGLA and EG2 driven by P*cc* could led to improvement of the activities of BGL and EG, respectively, and both of them resulted in the FPA augmentation, a hypothesis was proposed that construction of strains co-overexpressing BGLA and EG2 directed by P*cc* could further optimize the *T. reesei* enzymatic system to improve the cellulolytic ability. To this end, the expression cassette of *bglA* driven by P*cc* (P*cc*-*bglA*-*ptrA*) was transformed to EG2-overexpression strain QPE15 using *ptrA* as the selectable marker gene. Afterwards, two transformants QPEB29 and QPEB70 were selected and verified to successfully express *bglA* (data not shown). Then, QPEB29 and QPEB70 were cultured in CPM for 7 days at 30 °C for cellulase production. QPEB29 and QPEB70 showed 2.4 and 2.2 IU/mL filter paper activities at the end of fermentation, respectively, which were increased by 178.1% and 156.5% in comparison to QP4, and were also higher than that of QPB55 and QPE15 as expected (Fig. 7). These results indicated that the double overexpression of BGLA and EG2 directed by P*cc* could generate a more efficient cellulolytic enzyme system than the overexpression of the individual ones in *T. reesei*.

### Saccharification of corncob residues by the *T. reesei* strains co-overexpressing BGLA and EG2 directed by P*cc*

**Fig. 8:**
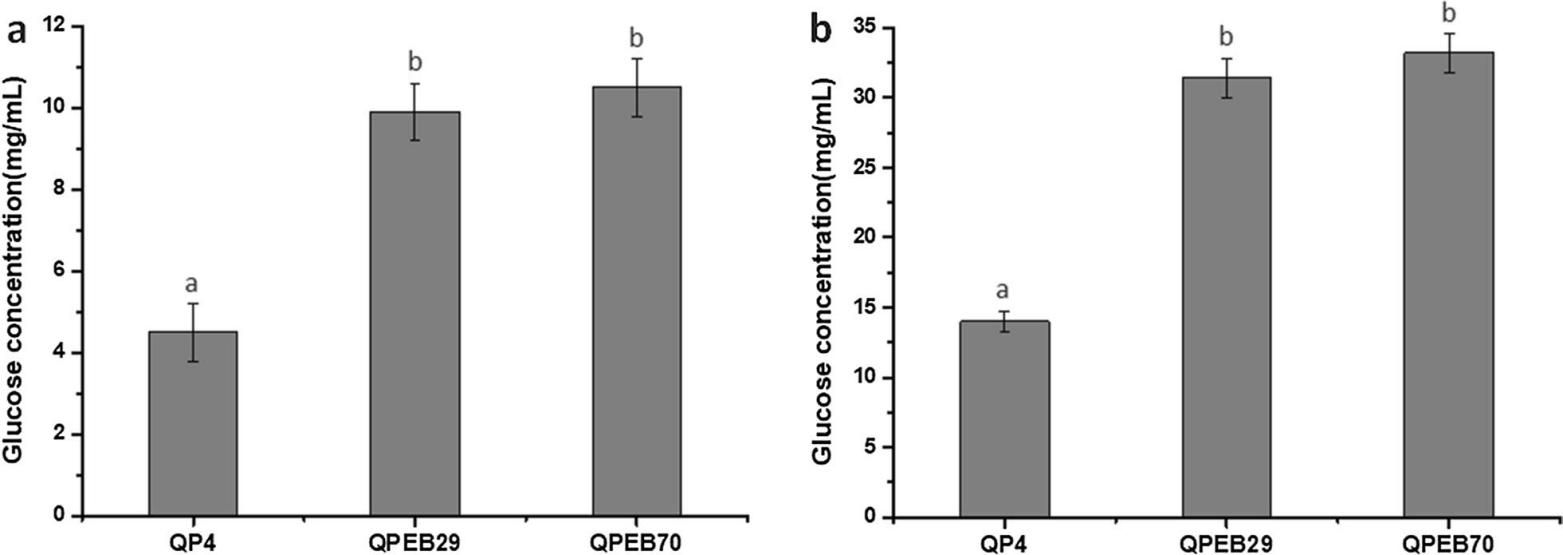
Saccharification of different pretreated corncob residues using the extracellular enzymes from QP4 and the transformants co-overexpressing *bglA* and *eg2* driven by P*cc*. **a** Glucose released from acid-pretreated corncob residue at 48 h. **b** Glucose released from delignified corncob residue at 48 h. Data indicates the mean of three independent experiments; error bars express the standard deviations. Different lowercase letters above the bars indicate significant differences at P < 0.05

The improved total cellulase activities of QPEB29 and QPEB70 led to the presumption that their extracellular cellulase cocktails might perform better than that of the parental strain in the deconstruction of lignocellulosic biomass. To confirm this, the cellulase cocktails produced by the *bglA*-*eg2* co-overexpression strains QPEB29 and QPEB70 were used in the saccharification of acid-pretreated corncob residue (ACR) and delignified corncob residue (DCR), respectively. Glucose released in saccharification towards ACR by cellulase cocktails of QPEB29 and QPEB70 were 9.9 ~ 10.5 mg/mL (corresponding to 28.5 ~ 30.2% cellulose conversion), which were 2.2- and 2.3-fold higher than that of QP4 (4.5 mg/mL, corresponding to 12.9% cellulose conversion, Fig. 8a). When DCR was used as the substrate, the released glucose detected in the saccharification with QPEB29 and QPEB70 cellulase cocktails reached 31.4 ~ 33.2 mg/mL (corresponding to 86.0 ~ 91.0% cellulose conversion), which were 2.2- and 2.4-fold higher than the value of QP4 (14.0 mg/mL, corresponding to 38.4% cellulose conversion, Fig. 8b). These results demonstrated that the double overexpression of BGLA and EG2 directed by P*cc* significantly improved the capacity of the *T. reesei* cellulase system for biomass hydrolysis, implying that this hybrid promoter P*cc* has the potential to be utilized as a robust promoter to optimize cellulolytic enzyme system for highly efficient bioconversion of lignocellulosic biomass.

## Discussion

In *T. reesei*, cost-effective cellulolytic enzyme system is needed to achieve efficient bioconversion of lignocellulosic biomass, and it could be obtained by modifying strains through improving cellulase expression levels and optimizing enzyme system [29, 30]. Promoter development is an essential method for strain modification and metabolic engineering [9]. Thus, this study created a novel hybrid promoter P*cc* using the synthetic approach in *T. reesei*, which enabled higher gene expression than that of the powerful promoter P*cbh1* and P*cdna1*.

As there is no software to predict promoter function in *T. reesei* as yet [10], extensive experiments were needed to investigate the ability of promoters. Here, one of the *T. reesei* strong constitutive promoters P*cdna1* was truncated from 5’ end to characterize its function. The deletion of a 154-bp sequence (− 794 to − 640 bp) between P*cdna1-2* and P*cdna1-3* resulted in an augment (74% with glucose and 300% with Avicel) of promoter activity (Fig. 2b and c). It was speculated that the binding sites of transcriptional repressors may exist between − 794 to − 640 bp, whose effect on inhibition of gene expression was even stronger than the activation effect of GC box located in this sequence. Besides, deficiency of the 150-bp sequence (− 640 to − 490 bp) between P*cdna1-3* and P*cdna1-4* generated a conspicuous reduction (23% with glucose and 59% with Avicel) of promoter activity (Fig. 2b and c). Indeed, a CCAAT box is contained in this fragment, and the lack of this *cis*-element might be responsible for the reduced gene expression intensity. It was reported that the modified *cbh1* promoter replacing three Cre1-binding sites to one activator Ace2-binding site and two CCAAT boxes showed 5.5-fold increase of gene expression while the promoter replacing a Cre1-binding site to an Ace2-binding site only exhibited a 1.9-fold increase [21]. In addition, disruption of AAB1, a homolog of Hap5 binding to CCAAT box, enabled an approximately 50% reduction of *am* gene expression in *Neurospora crassa* [39]. These reports indicated that the CCAAT box is an important motif in facilitating gene expression. As a result, it is likely that the absence of the CCAAT box in the promoter P*cdna1-4* resulted in the depressed promoter strength in this study. Nevertheless, it is also possible that uncertain enhancers exist in the 150-bp region (-640 to -490 bp) between P*cdna1-3* and P*cdna1-4*. In addition, chromatin accessibility is another essential factor to regulate gene expression (Klemm et al*.* 2019). In this study, the deficiency of the two fragments between P*cdna1-2* and P*cdna1-4* may have changed the structure of the chromatin, reduced the number of binding sites of the transcriptional factors that can directly binding to the nucleosomal DNA, or may have altered the sequence of nucleosome free region, in this case, the DNA accessibility of P*cdna1* may have been changed and thus significantly regulated the gene expression. Collectively, the promoter P*cdna1-3* containing CCAAT box and maybe unknown enhancers can strongly initiate gene expression and serve as a strong promoter.

In the regulation regions of promoters, there exist different transcription factor-binding sites, which comprise of activation sequences and repression sequences [40]. And alteration of these *cis*-elements would enable diversification of promoter activity [21, 22]. As the strongest inducible promoter in *T. reesei*, P*cbh1* was widely used in protein production and was well characterized [16, 29, 41]. Here, the 200-bp modified activation region of P*cbh1*, which contains the binding sites of Xyr1, Ace2 and Hap 2/3/5 complex but lacks Cre1-binding sites region, was deleted in this study to test its magnitude of amplifying expression. The deficiency of this activation region led to a dramatically decrease in promoter activity, which suggested that this activation region was indispensable to P*cbh1*. Meanwhile, the P*cbh1* core region P*cbh1-cr* lacking this 200-bp activation region showed a low basal activity (Fig. 3b and c), which raised the possibility that the activity of P*cbh1* could be further improved by replacing the native low-activity core region to a highly efficient one.

Usually, a simple synthetic hybrid promoter requires a minimal core promoter and the defined enhancer [24]. It was found that the abilities of the core sequence and the enhancer of a hybrid promoter were mutually independent, however, both of their strength and combination contribute to the capability of the hybrid promoter [25]. Indeed, based on the strong constitutive promoter P_GPD_, a hybrid promoter has been constructed in *Saccharomyces cerevisiae* using P_GPD_ as the core region and the upstream activation sequence (UAS) tandem of P_CLB_ as the activation region, expanding the transcriptional ability of P_GPD_ by 2.5-fold [26]. It can be concluded that a constitutive promoter could be used as the core region in a hybrid promoter, and in doing so, the promoter activity might be further improved. In this study, to acquire a powerful hybrid promoter, the high-efficient region of the strong *T. reesei* constitutive promoter P*cdna1* was selected as the core promoter and the regulation region of P*cbh1* was modified to serve as the enhancer. The hybrid promoter P*cc* using the high-efficiency promoter P*cdna1-3* as core region and the 200-bp (− 821 to − 622 bp) modified region of P*cbh1* as activation region exhibited an improved protein expression capacity compared with P*cbh1* and P*cdna1* under Avicel condition, indicating that the facilitation of the P*cbh1* activation region was imparted successfully to the P*cdna1* core promoter. However, when glucose was used as the sole carbon source, P*cc* appeared comparable performance to P*cdna1*, which might owe to the *cis*-elements in the modified activation region of P*cc* that could not respond to glucose. It has been reported that not all of the promoters with extra UAS elements showed stronger activity in *Saccharomyces cerevisiae*, especially when more than two UAS elements were added [42]. In addition, Kiesenhofer et al*.* demonstrated that the inappropriate arrangements and distances of *cis*-elements have the significant impact on P*cbh1* strength [43]. In this study, the modified activation region containing three more CCAAT boxes inserted into the upstream of P*cdna1* could be regarded as new arrangements of *cis*-elements, which presumably caused no increase of promoter activity under glucose condition. Nevertheless, high expression of protein was achieved by using this hybrid promoter under Avicel condition, which suggested that P*cc* would be utilized to enable cellulase optimization in *T. reesei*.

BGLA of *A. niger* was found to be more efficient in enzymatic hydrolysis of cellulose compared with the native BGL1 in *T. reesei* on account of its lower adsorption onto lignin [44, 45]. As a result, this study overexpressed *bglA* of *A. niger* under the control of P*cc* in *T. reesei* to enhance its cellulose-hydrolyzing ability, which led to a 43.5- fold enhancement of BGL activity (27.2 IU/mL) and 85.2% of FPA (1.6 IU/mL) compared to the parental strain. It has been reported that 3.7-fold increased BGL activity was obtained by overexpressing *bgl1* using a four-copy *cbh1* promoter [32]. Besides, a modified *cbh1* promoter replacing three Cre1-binding sites to two binding sites of transcriptional activator Ace2 and one of Hap2/3/5 complex was also used to overexpress *bgl1*, which resulted in a 27-fold augment of BGL activity (19 IU/mL) and 11% of FPA compared to the parental strain RUT-C30 [11]. Here, the transformants expressing the *A. niger* BGLA driven by P*cc* showed higher β-glucosidase activity than the strains in these previous studies, indicating that this chimeric promoter P*cc* exhibited prominent *bglA*-expression capacity. Furthermore, when the transformants were cultured under glucose condition, BGLA could also be detected in a high level with pure extracellular background, which provided an easier and low-cost method to purify BGLA when necessary.

In addition, the EG activity had a 20% augment after overexpressing *eg2* using P*cc* in *T. reesei*, and FPA was increased by 145% under inducible condition. These results were in agreement with the previous report that the EG2-deletion stain showed an absence of the expression of the other cellulase genes, demonstrating that EG2 plays a pivotal role in forming the inducer of cellulase to hydrolyze cellulose in *T. reesei* [46]. Collectively, overexpression of EG2 directed by P*cc* successfully optimized the cellulolytic enzyme system of *T. reesei.* Besides, when transformants cultured under glucose condition, EG2 was also secreted with pure extracellular background (Fig. 6a), which implied that P*cc* could also be utilized to simplify and cost down the process to purify the cellulases in *T. reesei*.

In accordance with the previous report that the overexpression of BGL from *Penicillium decumbens* led to an eightfold higher BGL activity but only a 30% increase of FPA [29], the 43.5-fold enhancement of BGL activity in this study only resulted in a 90% improvement of FPA. Nevertheless, the 20% increase of EG2 resulted in a 2.5-fold FPA improvement. These results prompted us to construct strains co-overexpressing BGLA and EG2 activated by P*cc* to further improve the enzymatic hydrolysis capability. As expected, the BGLA-EG2 double-overexpressed strain exhibited the significant enhancement in total enzyme activity and biomass saccharification efficiency towards differently pretreated corncob residues, especially against ACR (increased by 130%), compared to QP4. In comparison, the P*cbh1*-drived overexpression of BGL derived from *A. niger* and *P. decumbens* enabled approximately 20% enhancement of saccharification efficiency using corncob residue and cornstalk as the substrate, respectively [29, 33]. Additionally, the overexpression of BGL1 directed by a modified four-copy promoter of P*cbh1* led to a 29% augment of saccharification efficiency towards corncob residue [32]. Previously, Szijártó et al*.* identified that EG2 acted as a key component in liquefying pretreated wheat straw [47]. Collectively, the transformants co-overexpressing *bglA* and *eg2* driven by P*cc* in this study exhibited the extraordinary saccharification performance compared to that of the strains individually expressing BGL, implying that EG2 might play an important role in liquefaction of pretreated corncob residues as well.

Given the results that the strains expressing proteins directed by P*cc* in this study did not show growth defects, it seems likely the transcription factors were not depleted and this promoter was accommodating to the cellular context in *T. reesei*. Thus, there would be a dose-dependent effect if extra P*cbh1*-AR fragments are appended 5’ to the P*cdna1-3* fragment, and it is also possible to tandemly add binding sites of transcriptional activators to the upstream of the promoter to construct more powerful hybrid promoters. Besides, the hybrid promoter strategy raises the possibility of creating desired fine-tuned promoters by combining different enhancers and core promoters with varied magnitudes of strength. Moreover, this synthetic hybrid promoter approach offered a generic strategy to improve cellulolytic enzyme system in bioconversion of lignocellulosic biomass.

## Conclusions

In this work, a synthetic hybrid promoter P*cc* was created and confirmed to direct high-level expression of the essential cellulase components BGLA and EG2 to achieve the optimized cellulase system in *T. reesei*. Furthermore, when applied to the saccharification of differently pretreated corncob residues, this cellulolytic enzyme system exhibited the prominent performance that displayed 2.3-fold higher saccharification efficiency than that of the parental strain. The chimeric promoter P*cc* constructed here was the first hybrid promoter applied in *T. reesei*, which expanded the promoter engineering toolbox in *T. reesei* and raised the possibility to generate powerful tunable promoters.

## Materials and methods

### Strains, plasmids, cultural conditions and enzyme preparation

*Trichoderma reesei* QM53, a strain lacking the *mus53* gene with high targeted integration frequency derived from QM9414 [48], was used as the host strain in characterization of a series of 5’ truncated promoters of *cdna1*. The uracil auxotrophic strain *T. reesei* QP4, also constructed from *T. reesei* QM9414 [49], was used as the control strain for other assays. The plasmids pIG1783 containing the enhanced green fluorescent protein-encoding gene (*egfp*), pAN7-1 containing *trpC* terminator (T*trpC*) and pME2892 containing the *ptrA* selectable marker gene were employed for gene fragments cloning [50–52]. The plasmid pAB4-1 carriying the *pyrG* selectable marker gene was adopted for genetic transformation [53]. The fungal strains were cultivated on potato dextrose agar (PDA) plates containing peeled potato extract 200 g/L, glucose (2%, w/v), agar (2%, w/v, Dingguo Corp., Beijing, China; GA010-500 g) for 7 days at 30 °C to harvest conidia. Then, 10^8^ conidia were inoculated in a 500 mL flask (200 rpm) containing 100 mL liquid glucose-minimal medium (GMM; MM, [54]) or Avicel-minimal medium (AMM) and incubated at 30 °C. The AMM medium contained 2% (w/v) Avicel substituted for 2% glucose as the sole carbon source and the remaining components of GMM. To produce cellulase, conidia (10^6^/mL) were first inoculated in a 500 mL flask containing 100 mL liquid MM (1% glucose) and incubated at 180 rpm and 30 °C for 36 h to prepare the seed culture. After that, 10 mL of the inoculum was transferred into 100 mL of the cellulase production medium (CPM, [55]) in a 500 mL flask at 200 rpm and 30 °C. The glucose- and CMC-esculin plates were used to screen the transformants showing β-glucosidase activity. The medium composition was as follows: 2.0 g/L (NH4)2SO4, 0.5 g/L MgSO4·7H2O, 1 g/L KH2PO4, 1.0 g/L yeast extract, 2‰ (v/v) Triton X-100, 3.0 g/L esculin, 0.5 g/L ferric citrate, 20.0 g/L agar, and 10.0 g/L glucose or 10.0 g/L sodium carboxymethyl cellulose (CMC-Na) was contained as the sole carbon source for glucose-esculin plate or CMC-esculin plate, respectively. The culture medium was supplemented with 0.1% uracil (Sigma, USA) in the entire research process when necessary.

### Construction of strains characterizing P*cdna1* and the activation region of P*cbh1*

In this study, expression cassettes were constructed with the method of Double-joint PCR [56]. Primers used in this study were listed in Table 1. Phanta® Super-Fidelity DNA Polymerase (Vazyme Biotech Co., Ltd., Nanjing, China) was used for PCR amplification. To construct the cassettes that a series of 5’ truncated *cdna1* promoters expressed *cbh1*, chromosomal DNA of *T. reesei* QM53 was used as the template to amplify five different lengths of 5’ deleted P*cdna1* using primer pairs cdna1-1UF (hphR) to cdna1-5UF (hphR)/cdna1-UR (cbh1F), respectively. Upstream homologous arm of *cbh1* was amplified using primer pair cbh1-UF/cbh1-UR (hphF), and downstream homologous arm *cbh1*-ORF was amplified using cbh1-F/cbh1-R. The selectable marker hygromycin B gene (*hph*) fragment was amplified by hph-F/hph-R. Then the five differently truncated promoters of *cdna1* were respectively employed to fuse with the upstream homologous arm of *cbh1*, *hph* and *cbh1*-ORF through Double-joint PCR, and primer pair cbh1-UF/cbh1-R was used to amplify the final cassettes (Fig. 2a). The expression cassettes were purified and transformed respectively into the protoplasts of *T. reesei* QM53 through the PEG-mediated transformation, which was described previously [54]. The transformants were screened on solid MM containing 300 μg/mL hygromycin B.

**Table 1:** Primers used in this study

Primers	Nucleotide sequence (5′ to 3′)
cdna1-1UF (hphR)	CCGTCACCAGCCCTGGGTTGCAGACAATGATGGTAGCAGCG
cdna1-2UF (hphR)	CCGTCACCAGCCCTGGGTTGCTCAAACAGCCTCATGGTTCCCAGA
cdna1-3UF (hphR)	CCGTCACCAGCCCTGGGTTGGCCGATTCCTTTCCCGGGATGCTCC
cdna1-4UF (hphR)	CCGTCACCAGCCCTGGGTTGTAGCGAGCGGCCATTTGGAGGTCAT
cdna1-5UF (hphR)	CCGTCACCAGCCCTGGGTTGGCTTGGGTTGGAAGCGATTCCAGGT
cdna1-UR (cbh1F)	ATGACGGCCAACTTCCGATACATGTTGAGAGAAGTTGTTGGATTGAT
cbh1-UF	TACGCCACTGTGAGGAGGCC
cbh1-UR (hphF)	TTCAATATCAGTTAAGGTCGTCGGTGAGCCACGTGCTTTTT
cbh1-F	ATGTATCGGAAGTTGGCCGTCAT
cbh1-R	TTACAGGCACTGAGAGTAGTAAGG
hph-F	CGACCTTAACTGATATTGAA
hph-R	CAACCCAGGGCTGGTGACGG
*cbh1*-1307UF	AAAGCGTTCCGTCGCAGTAG
*cbh1*-724UR	AAGCCGTTGGCAAATTAC
*cbh1*-677UF (cbh1-724UR)	AAGCCGTTGGCAAATTACCTTCACTCAGTCCAATCTCA
cbh1-UR (egfpF)	CTCGCCCTTGCTCACCATGATGCGCAGTCCGCGGTT
cbh1c-UF	GGTGAAGTGAAAGAAGACAGAGGT
cbh1-869UF	ACCTGTAAAGCCGCAATG
egfp-F	ATGGTGAGCAAGGGCGAG
egfp-R (TtrpCS)	TTTGATGATTTCAGTAACGTTAAGTTTACTTGTACAGCTCGTCCA
TtrpC-S	ACTTAACGTTACTGAAATCATCAAA
TtrpC-A	GAGTGGAGATGTGGAGTGGG
cbh1-621UR	GGAACAAACAAGCGACCC
cdna1-3UF (cbh1-621UR)	GGGTCGCTTGTTTGTTCCGCCGATTCCTTTCCCGGGATGCTCC
cdna1-1UF	CAGACAATGATGGTAGCAGCG
cdna1-UR (cbh1sp)	AGCACGAGCTGTGGCCAAGAAGGCCGAGATGACGGCCAACTTCCGATACATGTTGAGAGAAGTTGTTGGATTGAT
cdna1-UR (egfpF)	CTCGCCCTTGCTCACCATGTTGAGAGAAGTTGTTGGATTGATC
bglA-F (cbh1spR)	CTTGGCCACAGCTCGTGCTGTACGTGCCGTCACTTCCTT
bglA-R (Txyn1S)	CAACATCAACAGAACCTAGTTGCTTTAGTGAACAGTAGGCAGAGACG
eg2-F (cbh1spR)	CTTGGCCACAGCTCGTGCTCAGCAGACTGTCTGGGGCC
eg2-R (Txyn1S)	CAACATCAACAGAACCTAGTTGCTCTACTTTCTTGCGAGACACGA
Txyn1-S	AGCAACTAGGTTCTGTTGATGTTG
Txyn1-A	GAATAGCCAGAGGACGGTTG
ptrA-S	AACAAAGATGCAAGAGCGG
ptrA-A (cbh1-869UF)	CCGCTCTTGCATCTTTGTTACCTGTAAAGCCGCAATG

The cassettes expressing *egfp* directed by P*cbh1-dc* and P*cbh1-cr* were constructed by Double-joint PCR. Firstly, to delete the Cre1-binding sites region, two fragments ahead and behind this region were amplified by primer pairs cbh1-1307UF/cbh1-724UR and cbh1-677UF (cbh1-724UR)/cbh1-UR (egfpF), respectively. Secondly, after these two fragments were fused together, the P*cbh1-dc* sequence could be amplified using the fused product as the template and cbh1-869UF/cbh1-UR (egfpF) as primer pair. Then, *T. reesei* QP4 genome was used as the template to amplify P*cbh1-cr* by primer pair cbh1c-UF/cbh1-UR (egfpF). At the same time, the fragments *egfp* and *trpC* terminator were amplified from plasmid pIG1783 and pAN7-1 by prime pairs egfp-F/egfp-R (TtrpCS) and TtrpC-S/TtrpC-A, respectively.Next, the promoter gene fragments P*cbh-dc* and P*cbh1-cr* were respectively fused with *egfp* and *trpC* terminator through Double-joint PCR. Primer pairs cbh1-869UF/ TtrpC-A and cbh1c-UF/ TtrpC-A were used to construct the final cassettes P*cbh1-dc-egfp* and P*cbh1-cr-egfp*, respectively (Fig. 3a). Finally, the expression cassettes were purified and transformed respectively into the protoplasts of *T. reesei* QP4 together with the plasmid pAB4-1 through the PEG-mediated transformation strategy mentioned above. The transformants were screened on solid MM without pepton.

### Generation of promoter P*cc* and construction of the strains identifying P*cc*

The promoter P*cc* was constructed as follows. Firstly, P*cbh1-dc* was used as the template to amplify the activation region (AR) of P*cbh1* using primer pair cbh1-869UF/cbh1-621UR. Then, P*cdna1-3* was amplified by primer pair cdna1-3UF (cbh1-621UR)/cdna1-UR (egfpF). Subsequently, AR of P*cbh1* was fused to P*cdna1-3* and primer pair cbh1-869UF/cdna1-UR (egfpF) was used to finally amplify the P*cc* promoter (Fig. 4a).

After that, P*cc* was fused with *egfp* and T*trpC* to get the final expression cassette P*cc-egfp*. At the same time, P*cdna1-egfp* and P*cbh1-egfp* were constructed as control. P*cdna1* was amplified by primer pair cdna1-1UF/cdna1-UR (egfpF) and then fused with *egfp* and T*trpC* to get the expression cassette P*cdna1-egfp*. P*cbh1* was amplified by primer pair cbh1-869UF /cbh1-UR (egfpF) and then fused with *egfp* and T*trpC* to get the expression cassette P*cbh1-egfp*. Afterwards, these three cassettes were purified and respectively co-transformed with pAB4-1 into the protoplasts of *T. reesei* QP4 through the PEG-mediated transformation method mentioned above. The transformants were screened on solid MM without pepton.

### Construction of strains overexpressing BGLA or/and EG2 directed by P*cc*

To construct the BGLA and EG2 overexpression strains directed by P*cc* respectively, the expression cassettes were constructed as follows. The promoter P*cc* was amplified by primer pair cbh1-869UF/ cdna1-UR (cbh1sp) using P*cc* promoter constructed above as the template. Then the *bglA* (GenBank Accession No. AM270402) and *eg2* (protein ID: Tr_120312) genes were cloned by primer pairs bglA-F (cbh1spR)/bglA-R (Txyn1S) and eg2-F (cbh1spR)/eg2-R (Txyn1S), respectively. Chromosomal DNA of *A. niger* was used as the template to clone the β-glucosidase-encoding gene *bglA*. At the same time, the terminator T*xyn1* was amplified using the template QP4 genome and primer pair Txyn1-S/Txyn1-A. After that, the three fragments, P*cc*, *bglA*/*eg2* and T*xyn1* were fused together by Double-joint PCR to get the *bglA* and *eg2* overexpression cassettes, respectively. Finally, the cassettes were purified and transformed together with pAB4-1 respectively into the protoplasts of *T. reesei* QP4 through the PEG-mediated transformation method mentioned above. The transformants were screened on solid MM without pepton.

The BGLA and EG2 co-overexpression strains were constructed by transforming a P*cc*-*bglA*-*ptrA* cassette to QPE15. The *ptrA* gene fragment was cloned using the template pME2892 by primer pair ptrA-S /ptrA-A (cbh1-869UF). And the final co-overexpression cassette was constructed by fusing *ptrA* gene with BGLA overexpression cassette constructed above. The transformants were screened on solid MM with 2 μg/ml of pyrithiamine.

### Enzyme activity and SDS‑PAGE assay

The filter paper activity (FPA), cellobiohydrolase (CBH), β-glucosidase (BGL), and endoglucanase (EG) activities were measured using Whatman no. 1 paper (Whatman, UK), *p*-nitrophenyl-β-D-cellobioside (pNPC; Sigma, USA), *p*-nitrophenyl-β-D-glucopyranoside (pNPG; Sigma, USA) and CMC-Na (Sigma, USA) as substrates respectively by the methods described previously [57]. One unit of enzyme activity was defined as the amount of enzyme required to liberate one micromole reducing sugars (FPA, EG) or *p*-nitrophenol (CBH, BGL) per minute under the assay conditions. SDS-PAGE electrophoresis was performed in 12% polyacrylamide separating gel with equal volume of culture supernatants boiled for 10 min for degeneration. The specific cellulase bands were excised for MALDI-TOF–MS identification.

### Saccharification of corncob residues

Acid-pretreated (ACR) and delignified (DCR) corncob residues were used as the substrate in saccharification, and the components of these substrates had been described by Liu et al*.* [58]. The cellulase loading was 10 IU (FPA)/g substrate equally. The fermentation broths containing 5% (w/v) of corncob residue as the substrate were loaded to 30 mL by adding pH 4.8 citric acid buffer. The 30 mL reagent was placed in a 100 mL flask at 150 rpm and 50 °C for 48 h. Glucose production was detected with an SBA-40C biological sensor analyzer (BISAS, Shandong, China) at the end of saccharification.

### Fluorescence and light microscope

Fluorescence and light microscope (Nikon Eclipse 80i fluorescence microscope) was used to observe the eGFP expression of mycelia. 10^8^ spores of the eGFP-expression transformants were inoculated on GMM or AMM plates (GMM or AMM with 4 g/L agar) and cultured at 30 °C for 1 or 4 days. The coverslips were obliquely inserted in the plates at a proper distant from the spores, and the mycelia grown on coverslips was observed directly at the end of cultivation.

### Fluorescence intensity measurement

10 mg mycelia of the eGFP expression transformant cultured in GMM or AMM was added evenly to 200 µL distilled water and then transferred to a 96-well plate. The 96-well plate was scanned in a Victor2™ 1420 Multilabel Counter (Perkin Elmer, Boston, MA) with 485 nm excitation and 535 nm emission wavelengths to get the fluorescence intensity.

## Supplementary Information

**Additional file 1: Table S1**. Description of Cis-elements.

## Data Availability

The data generated or analyzed during this study are included in this published article and its additional information files. Further datasets used and analyzed during the current study are available from the corresponding author on reasonable request.

## Abbreviations

ACR: Acid-pretreated corncob residue
AMM: Avicel-minimal medium
BGL: β-Glucosidase
CBH: Cellobiohydrolase
CCR: Carbon catabolite repression
CMC-Na: Sodium carboxymethyl cellulose
CPM: Cellulase production medium
DCR: Delignified corncob residue
EG: Endoglucanase
FPA: Filter paper activity
GMM: Glucose-minimal medium
pNPC: P-Nitrophenyl-β-D-cellobioside
pNPG: 4-Nitrophenyl-β-dglucopyranoside
SDS-PAGE: Sodium dodecyl sulfate polyacrylamide gel electrophoresis
UAS: Upstream activation sequence
