# Supplementary material for: Development of a powerful synthetic hybrid promoter to improve the cellulase system of Trichoderma reesei for efficient saccharification of corncob residues
Source: Microb Cell Fact. 2022 Jan 4;21:5. doi: 10.1186/s12934-021-01727-8 (PMC8725555; doi:10.1186/s12934-021-01727-8)
Supplement: Supplementary file 1 — Additional file 1: Table S1. Description of Cis-elements. [file 12934_2021_1727_MOESM1_ESM.docx]

**Table S1. Description of *Cis*-elements**

| ***Cis*-element** | **Description** |
| --- | --- |
| CCAAT box (Hap 2/3/5-binding site) | Common *cis*-acting element in promoter of numerous eukaryotic genes, a binding site of the transcriptional activator Hap 2/3/5 |
| TATA box | Core promoter element |
| GC box | Enhancer-like element |
| Xyr1-binding site | A binding site of the transcriptional activator Xyr1 |
| Ace2-binding site | A binding site of the transcriptional activator Ace2 |
| Cre1-binding site | A binding site of the carbon catabolite repressor Cre1 |
